# Immunopotentiating effects of herb-partitioned moxibustion on the spleens of cyclophosphamide-induced immunosuppressed rats

**DOI:** 10.1186/s13020-024-00898-x

**Published:** 2024-02-18

**Authors:** Luojie Xiong, Yuefeng Tian, Xiaoshan Xu, Huifang Wang, Wei Li, Chuntao Zhai

**Affiliations:** 1 College of Acupuncture, Massage and Rehabilitation, Hunan University of Chinese Medicine, Changsha, 410208 China; 2 Second Clinical College, Shanxi University of Chinese Medicine, Jinzhong, 030619 People’s Republic of China

**Keywords:** Herbal-partitioned moxibustion, Immunosuppression, CD28, CTLA-4

## Abstract

**Background:**

To investigate the effec of the herb-partitioned moxibustion on T-lymphocyte activity in immunosuppressed rats through differential modulation of the immune checkpoint molecules CD28 and CTLA-4.

**Methods:**

Forty-eight Sprague‒Dawley rats were randomly divided into the normal group (NG), the cyclophosphamide model group (CTX), the herb-partitioned moxibustion group (HPM), the CD28 inhibitor + herb-partitioned moxibustion group (aCD28 + HPM), the CTLA-4 inhibitor + herb-partitioned moxibustion group (aCTLA-4 + HPM), and the levamisole group (LEV) (8 rats per group). The immunosuppression model was prepared using cyclophosphamide. HPM treatments was performed via herb-partitioned moxibustion at 4 acupoints, Zhongwan (CV12), Shenque (CV8), Guanyuan (CV4), and Zusanli (ST36). Subsequently, the moxa floss was made into a conical moxa cone, which was then placed on the herbal cake and ignited. Five consecutive moxibustion strokes were performed daily for 10 consecutive days. In addition to the same moxibustion, each rat in the aCD28 + HPM group was injected intraperitoneally with 0.5 mg/kg of CD28 inhibitor per rat on the first day of treatment, and 100 μL of CTLA-4 inhibitor was injected into the aCTLA-4 + HPM group on Days 1, 4, and 7. For the positive control, levamisole (LEV) was administered by gavage at a dose of 2 mg/kg once daily for 10 days.

**Results:**

Compared with those in CTX model rats, the WBC counts in the HPM and other groups were significantly higher. The immobility time of EPM in the HPM group was significantly lower than that of the CTX group. The HE stainin results also showed that after treatment, the the marginal zone area of the spleen tissue in the HPM increased, the number of lymphatic sheath lymphocytes around the small central artery of the spleen increased, and the amount of red pulp containing a small amount of pigmentation was partially reduced. Compared with those in the CTX group, the serum levels of CD28, CTLA-4, B7-1, and B7-2 were significantly lower, and the levels of α-MSH, TrkB, and BDNF were significantly greater in the HPM group. The results of the flow cytometry assay showed a significant increase in the number of CD8 + T lymphocytes after treatment with HPM or other agents compared to that in the CTX group. The immunofluorescence results showed that the levels of CD28 and CTLA-4 lower in spleen tissues than in control tissues, and the binding ability of CD28 to B7-1 and B7-2 was weakened after treatment with HPM and other treatments compared with CTX rats, PCR for CD28, CTLA-4 and B7-1 showed similar results.

**Conclusion:**

In the immunosuppressive rat model induced by cyclophosphamide, HPM upregulated the expression of α-MSH, TrkB, and BDNF, and downregulated the expression of CD28 and CTLA-4, thereby enhancing the activity of CD_8_^+^ T lymphocytes, restoring spleen function, improving the immunosuppressive state, restoring immune function, and effectively alleviating depressive symptoms.

## Introduction

Based on WHO statistics, the average global life expectancy was 73 years in 2019. It is predicted to reach 77 years in 2048. However, the mortality rate of patients with noncommunicable diseases will also increase from 73 to 86%. Noncommunicable diseases are a serious medical issues that aging societies must confront [1]. The primary cause of noncommunicable diseases resulting from aging is adverse health outcomes caused by weakened physical conditions and a compromised immune system [2]. The immune system plays a vital role in the prevention and treatment of noncommunicable diseases. When the body’s immune function is low or in an immunosuppressed state (suppression of the immune system’s ability to resist disease), the body’s defense capabilities are weakened [3, 4]. Typically, immunosuppressed patients exhibit a decrease in the number of active immune cells, abnormal secretion of related cytokines, and enhanced activity of immunosuppressive factors [5].

Cyclophosphamide, as an alkylating agent, is utilized as an immunosuppressive drug in the treatment of cancer [6]. Nevertheless, cyclophosphamide can also suppress the immune system by damaging cellular DNA, inhibiting the proliferation of splenic lymphocytes, and reducing cytokine secretion [7–9].

Lymphocytes play an important role in regulating immune suppression. The recovery of T-cell numbers can enhance the body’s immune function and correct the imbalance of immune suppression by restoring the levels of various cytokines [10, 11]. Immune checkpoints are a group of molecules expressed on immune cells that regulate immune activation. Recent studies have shown that tumor immunotherapy targeting immune checkpoints such as CTLA-4 and CD28, can render cancer cells incapable of resisting immune responses, thereby achieving the therapeutic objective of antitumor therapy [12]. CD28, a costimulatory immune checkpoint molecule, enhances the activation of T cells, thereby ameliorating immune suppression or exacerbating the progression of autoimmune diseases [13, 14]. CTLA-4, a coinhibitory immune checkpoint, typically exerts inhibitory effects on T cells [15]. Studies on CTLA-4 and CD28 in nontumor fields other than oncology have mainly concentrated on autoimmune diseases, transplantation, and other domains, with limited research on immunosuppression [16, 17].

Moxibustion has long been regarded as a health therapy that enhances the body’s immune function and is an important treatment method in Asian medicine. Recent studies have shown that moxibustion has antioxidant, anti-inflammatory, and immunomodulatory effects [18]. Herbal-partitioned moxibustion is performed with moxa cones made of refined mugwort floss, which are placed on Traditional Chinese medicinal formula-dispensing herbal cakes at acupoints to treat diseases [19]. Moxibustion is one of the traditional Chinese medicine treatment methods, and in several clinical studies, it has been proven that moxibustion can regulate the body’s immune function. Research has shown that indirect moxibustion treatment can increase the number of CD_3_^+^ and CD_4_^+^ T lymphocytes in normal individuals and reduce the percentage of CD_8_^+^ T cells in systemic lupus erythematosus patients [20]. Additionally, studies suggest that moxibustion can downregulate the levels of HIF-1α and VEGF, improving symptoms of rheumatoid arthritis [21]. Furthermore, a clinical study indicates that herb-partitioned moxibustion can downregulate the levels of IL-2 and IL-12 in patients with ulcerative colitis [22]. Nonetheless, high-quality clinical reports on the modulation of the human immune system by moxibustion are still lacking.

Our previous studies have shown that HPM treatment of acupoints (CV4, CV8, CV12, and ST36), which have been confirmed to enhance physical health by elevating IL-4, IL-1β, MyD88, and other factors [18, 23, 24], can improve immune suppression by modulating the PD-1 immune checkpoint and affecting ubiquitination, thereby promoting immune recovery [25, 26]. Nevertheless, preliminary results suggest that the ability of moxibustion to ameliorate immune suppression may involve multiple targets, and further studies are warranted.

In this study, we used an immunosuppressed rat model prepared with cyclophosphamide to investigate whether HPM could improve the locomotor behavior of immunocompromised animals, and we also evaluated whether HPM could ameliorate inflammatory responses. Furthermore, we attempted to analyze the colocalization of HPM with CD28 and CTLA-4 and their ligands in immune organs, aiming to elucidate the differential regulatory mechanisms of HPM on T lymphocytes through distinct immune checkpoints for the restoration of immune function.

## Materials and methods

### Reagents

Cyclophosphamide (22011925, Hengrui Pharmaceuticals Co., Ltd, Jiangsu, China); Levamisole (200201, Renhetang Pharmaceuticals Co., Ltd, Shandong, China); InVivoMAb recombinant CTLA-4-Ig (CD28 inhibitor, BE0099, BioXCell, USA); InVivoSIM anti-human CTLA-4 (CTLA-4 inhibitor, SIM0004, BioXCell, USA); Pentobarbital (211005, Changjiang Biological Laboratory Technology Development Co., Ltd, Shijiazhuang, China); eosin and hematoxylin (181024, LeXiang Medical Reagent Technology Co., Ltd, Shanghai, China); absolute ethanol, methanol, xylene, isopropanol (Zhiyuan Chemical Reagent Co., Ltd, Tianjin, China); Enzyme-linked immunosorbent assay (ELISA) kits were purchased from Meimian (Meibiao Biotechnology Co., Ltd, Jiangsu, China); anti-CD3-APC (B354716, BioLegend, USA), anti-CD4-FITC (B361172, BioLegend, USA) and anti-CD8-PE (B357604, BioLegend, USA); Sheath fluid (342003, BD, USA); TriQuick Reagent (R1100, Solarbio, Beijing, China); TransScript cDNA Synthesis SuperMix for qPCR (AT341, Solarbio, Beijing, China); PerfectStart Green qPCR SuperMix (AQ602, Transgen, Beijing, China); anti-CTLA-4 antibody (bm5388, Boster, USA); anti-CD28 antibody (ab243228, Abcam, USA); anti-B7-1 antibody (sc-376012, Santa Cruz, USA); anti-B7-2 antibody (sc-28347, Santa Cruz, USA); Cy3 Conjugated affinipure goat anti-mouse IgG (BA1031, Boster, USA).

### Animals and preparation of immunosuppressive models

Eight-week-old SD rats, half male, and half female, were used. The animal room was maintained on a 12-h light–dark cycle (lights on at 6 am). The rats were housed in a noise-free environment, and after one week in the animal facility, all rats were habituated to the experimenter handling the intraperitoneal injections and test chambers. All rat studies were approved by the Medical Ethics Committee of Shanxi University of Traditional Chinese Medicine (Ethics number 2021DW036). Food and tap water were available ad libitum.

Forty-eight rats were randomly assigned to the normal group, the cyclophosphamide model group, the herb-partitioned moxibustion group, the CD28 inhibitor + herb-partitioned moxibustion group, the CTLA-4 inhibitor + herb-partitioned moxibustion group or the positive control group using the complete random grouping method, with a total of six groups of eight rats each.

To formulate a solution of cyclophosphamide at a concentration of 20 mg/mL per vial, 0.2 g of cyclophosphamide powder was dissolved in 10 mL of 0.9% sodium chloride solution for each vial. The rats were weighed daily prior to the initiation of the modeling process. Following the methodology outlined by Zhang et al. [9], an immunosuppression model was induced by intraperitoneal injection of cyclophosphamide at a dosage of 40 mg/kg/day for three consecutive days across all the rat groups, excluding the normal group. The normal group, during the same time frame, received intraperitoneal injections of saline.

### Herbal-partitioned moxibustion (HPM) treatments

**Fig. 1:**
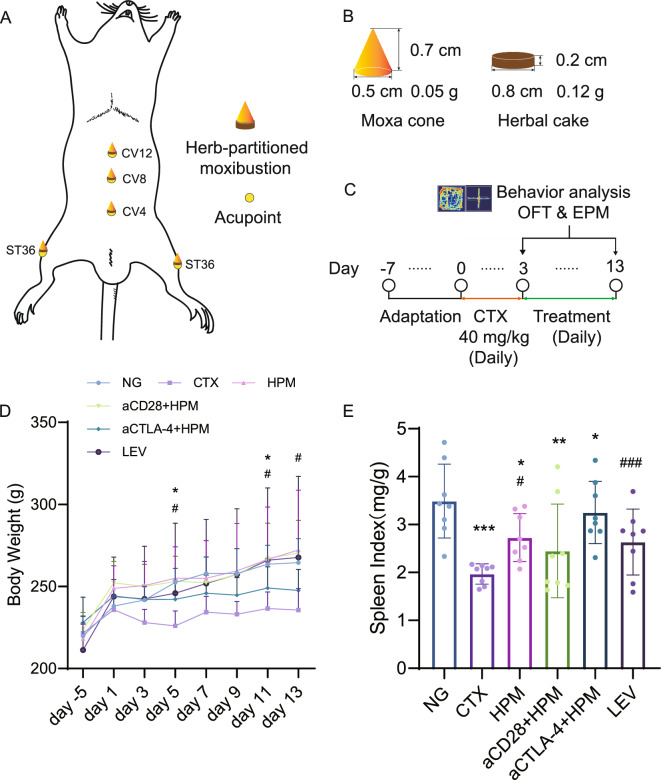
HPM treatment restored body weight and increased spleen index in immunosuppressed rats. **A** Schematic diagram of the acupoints used in this experiment. **B** Dimensional specifications of the HPM in the experiment. **C** Schematic showing timeline for CTX induction, HPM treatment and behaviour experiments. **D**, **E** Mean weight of each group (n = 8 per each group). Data are expressed as means ± SD, *One-way ANOVA* tests with *LSD-t* post-hoc tests executed. ^*^p < 0.05, ^**^p < 0.001, ^***^p < 0.001 vs NG; ^#^*p* < 0.05, ^###^*p* < 0.001 vs CTX. SD: standard deviation; ANOVA: analysis of variance; LSD-t: Least Significant Difference-t, NG: normal group; CTX: cyclophosphamide; HPM: Herb-partitioned moxibustion; aCD28: anti-CD28; aCTLA-4: anti-CTLA-4; LEV: Levamisole

The rats were treated with moxibustion at the acupoints of Guanyuan (CV4), Shenque (CV8), Zhongwan (CV12), and Zusanli (ST36) for the following 10 days (Fig. 1A, C). The following 4 acupoints were selected for their potential to improve immunity. Previous studies have shown that stimulating these factors can have a positive effect on the immune system [18, 23, 24, 27]. The location of moxibustion was determined according to the WHO standard acupoints as well as the anatomical location of previous rat acupoint [28] (Fig. 1A). During moxibustion across the herbal cake, the rats were immobilized on a rat plate and subsequently moxa cones (Fig. 1B) were placed on the herbal cake (Fig. 1B) and then placed on the acupoints, except for the ST36, which was treated bilaterally, while all the other acupoints were treated unilaterally. The moxa floss used to make moxa cones comes from TongRenTang (180826, TongRenTang Herbal Medicine Co., Ltd, Beijing, China).The average duration of moxibustion was at 3 min per moxa, and 5 moxa cones were applied consecutively daily for 10 days. The moxibustion operators are professionally trained in Chinese medicine techniques (License number: 241360203000015).

The aCD28 + HPM group will receive an intraperitoneal injection of 0.5 mg/kg CD28 inhibitor on the first day of treatment in addition to 10 days of HPM treatment [29].

In the aCTLA-4 + HPM group, 100 μL of an CTLA-4 inhibitor [30] was injected intraperitoneally on the first, fourth, and seventh days of treatment, and the rest of the treatments were the same as those used in the HPM group.

The positive controls were treated daily with levamisole solution configured at a dose of 2 mg/kg by gavage daily for 10 days [31]. As controls, all animals were immoobilized at the same time and all groups except the positive control group were gavaged with equal amounts of saline to ensure consistency of pressure between groups.

### Determination of body weight and spleen organ indices in rats

The rats were weighed at the end of the treatment and body weight data were recorded. The rats were subsequently sacrificed and anesthetized with 3% pentobarbital sodium intraperitoneally (1.5 mL/kg). Blood was collected, and the spleens were harvested and weighed. The spleen index was calculated by the following formula: The spleen index = spleen mass (mg)/body mass (g).

### Anxiety-like behavioral tests

We used the open-field test (OFT) and elevated plus-maze (EPM) test to evaluate the mood levels, especially the anxiety levels, in immunosuppressed model rats after the end of treatment (Fig. 1C).

#### Open-field test

For OFT, after 1 h of adaptation to the experimental environment, rats were individually housed in a black acrylic box (100 × 100 × 40 cm), and the total distance traveled, the distance to the central area, the ratio of central and total distance, and the number of times they crossed the central area in 5 min were then recorded with a camera running the OFT-100 program (Taimeng Software Co., Chengdu, China). At the end of the experiment for each rat, the feces and urine were removed from each rat, and the observation box was scrubbed with 75% ethanol to eliminate olfactory cues.

#### Elevated plus-maze

The EPM test was performed immediately after the rats completed the OFT experiment. After habituation for approximately 1 h, the rats were placed in the central area of the maze (arm length, 50 cm; arm width, 10 cm; closed arm height, 40 cm; ground clearance, 60 cm) in a quiet environment with their faces facing the open arm, and their movement data were recorded by a camera monitor vertically and by PMT-100 analysis system (Taimeng Software Co., Chengdu, China) for 5 min. The total distance traveled, the immobility time, the number of entries into the open arms (OE), and the ratio of the number of entries into the open arms (OE%) were used as evaluation indices. The cleanup method was the same as that for the OFT.

### Complete blood count (CBC)

One day after treatment ends, after anesthetized 2.5 mL of blood was collected from the abdominal aorta of the rats using an EDTA blood collection tube. The blood was shaken well to prevent coagulation and subsequently measured on a F580 (Maccura Biotechnology Co., Chengdu, China) for counts of WBC, NEU%, LYM%, MONO%, EOS%, BASO%, RBC, HGB, and HCT.

### Hematoxylin and eosin staining (H&E) stainning

In each group of rats, the spleen was exposed by opening the chest after anesthetized, and the spleen tissue was subsequently removed and fixed in 4% paraformaldehyde for 24–48 h. After conventional dehydration in gradient ethanol, paraffin embedding was performed. Finally, the paraffin-embedded tissue was sliced coronally into continuous sections with a thickness of 5 μm using a microtome, dried in an oven, and stored for use. The prepared paraffin-embedded sections were routinely dewaxed, hydrated, and immersed in hematoxylin staining solution for 5 min to observe the coloration under the microscope. Then, 0.5% hydrochloric acid ethanol was added to the mixture for 30 s, which was subsequently soaked in tap water for 15 min, rinsed with pure water, immersed in eosin staining solution for 2 min, and washed and sealed with neutral gum. Morphological and structural changes in the rat spleen tissue were observed under a 4× and 20× light microscopes.

### Enzyme-linked immunosorbent assay (ELISA)

Plasma levels of CD28, CTLA-4, B7-1, B7-2, α-MSH, TrkB, 5-HT, and BDNF were measured according to the instructions of the ELISA manufacturer for samples, standards, etc. The plate was read using a BioTek Epoch2 microplate reader (Molecular Devices, Bio Tek Instruments, CA, USA), and the absorbance was measured at a wavelength of 450 nm using Gen5 software version 3.08 (Molecular Devices). The corresponding standard curves were established to calculate the serum CD28, CTLA-4, B7-1, B7-2, α-MSH, TrkB, 5-HT, and BDNF levels.

### Flow cytometry

Flow cytometry was performed on single cell suspensions of splenocytes. A total of 1 × 10^6^ cells were resuspended in PBS containing 1% BSA and the fluorescently labeled specific antibody in the appropriate dilution. Our antibody panel includeed anti-CD3-APC, anti-CD4-FITC, and anti-CD8-PE. After shaking, the cells were incubated at 4 °C for 20 min in the dark. After the incubation, add 1 mL of PBS was added, the mixture was shaken and centrifuged for 5 min at 1500 rpm, after which supernatant was discarded; this process was repeated twice. Then, 300 μl of sheath fluid was added to each test tube, the tube was shaken well, and the cells were analyzed on a flow cytometer (BD FACS Celesta, USA).counted the distribution of 50,000 cells. The data were acquired from BD FACS Diva™ Software and analyzed using FlowJo version 10 (FlowJo Software, LCC, USA).

### Real-time quantitative PCR

After the treatment, we extracted total RNA from spleen tissues using TriQuick Reagent, and assessed RNA integrity through agarose gel electrophoresis. Subsequently, cDNA synthesis was performed via reverse transcription using the TransScript kit. The RNA was reverse transcribed and converted into cDNA using a TransScript kit; the resulting cDNA was subsequently amplified using the PerfectStart Green qPCR SuperMix. The PCR predenaturation temperature was set at 94 °C for 2 min, constituting one cycle. The cycling procedure involved denaturation at 94 °C for 5 s, followed by annealing and extension at 60 °C for 30 s, this process was repeated for a total of 45 cycles. The amplification results were computed using LightCycler480 software (Roche, GRE), and relative gene expression was determined using the 2^−ΔΔCt^ method. The sequences of primers uesd are provided in Table 1.

**Table 1:** Primer sequences

Gene	Forward primer	Reverse primer
CD28	5′GGGTGGAGGTAGGTTGGAGTA3′	5′AGGAAGTGAGGAAACAAGCCC3′
CTLA-4	5′CTTGCTGCAGTTAGTTCGGG3′	5′GGCTCTGTTGGGGGCATTTT3′
B7-1	5′AAGGGAAGTTGGAAAGGGGAAA3′	5′GAACCGAGGTCTTGAGCCTT3′
GAPDH	5′ACTCTACCCACGGCAAGTTC3′	5′TGGGTTTCCCGTTGATGACC3′

### Immunofluorescence

After HPM and inhibitor treatment, rat spleen tissue was collected, fixed in tissue fixative, subsequently embedded, and sectioned at room temperature (5 μm). The sections were incubated with primary antibodies against CTLA-4 (1:100), CD28 (1:100,), B7-1 (1:100) and B7-2 (1:100) overnight at 4 °C. The following day, the sections were incubated with secondary antibodies at room temperature for 1 h in the dark. The CTLA-4 and CD28 fluorescence intensities were calculated and the fluorescence colocalization of CTLA-4 bound to B7-1 and B7-2, and that of CD28 bound to B7-1 and B7-2 were calculated. Images and scatter plots were analyzed by a researcher using ImageJ 2.0 software (NIH, Bethesda, MD, USA). The points corresponding to CTLA-4 (green fluorescence) with B7-1 (red fluorescence), CTLA-4 with B7-2 (red fluorescence), CD28 (green fluorescence) with B7-1 (red fluorescence) and CD28 with B7-2 (red fluorescence) were generally concentrated on diagonal lines.

### Statistical analysis

Continuous variables with a normal distribution are presented as the means ± SDs; nonnormal variables are reported as medians with interquartile range. The data were analyzed using the software SPSS 23.0 software for Windows (SPSS, Inc., Chicago, IL, USA). The normality and homogeneity of variance of the datasets were checked by the Shapiro‒Wilk and Brown–Forsythe tests, respectively. *One-way analysis of variance (ANOVA)* was used to analyze the inter-group differences. For data that follows a normal distribution, the *least significant difference-t* (LSD-t) test was used for multiple comparisons if the variances are homogeneous. If the variances are heterogeneous, the *Dunnett’s T3* method was used. *Non-parametric* tests were used for data that did not follow a normal distribution. Differences were considered to be significant at a *p* value < 0.05 and were further stratified to *p* < 0.01 and *p* < 0.001.

## Results

### Effect of HPM treatment on body weight and spleen index in immunosuppressed rats

The body weights of the rats were measured daily. The body weight of the CTX rats decreased significantly beginning on day 5, and the trend toward a decrease changed significantly on days 5, 11, and 13. On days 5 and 13, the difference between the NG and CTX groups was significant (*p* = 0.016, *p* = 0.043), but on day 13, there was no significant difference between the two groups (*p* > 0.05). At the end of treatment, weight gain was significantly greater in the HPM (*p* = 0.006), aCD28 + HPM (*p* = 0.007), and LEV (*p* = 0.012) groups than in the CTX group (Fig. 1D).

In addition, the splenic indices were significantly lower in the CTX (*p* < 0.001), HPM (*p* = 0.030), aCD28 + HPM (*p* = 0.004) and aCTLA-4 + HPM (*p* = 0.015) groups than in the NG group. Compared with those of CTX, the the splenic indices of HPM (*p* = 0.030) and LEV (*p* < 0.001) were significantly higher (Fig. 1E).

### Effect of HPM in combination with inhibitor treatment on depressive-like behavior in immunosuppressed rats

**Fig. 2:**
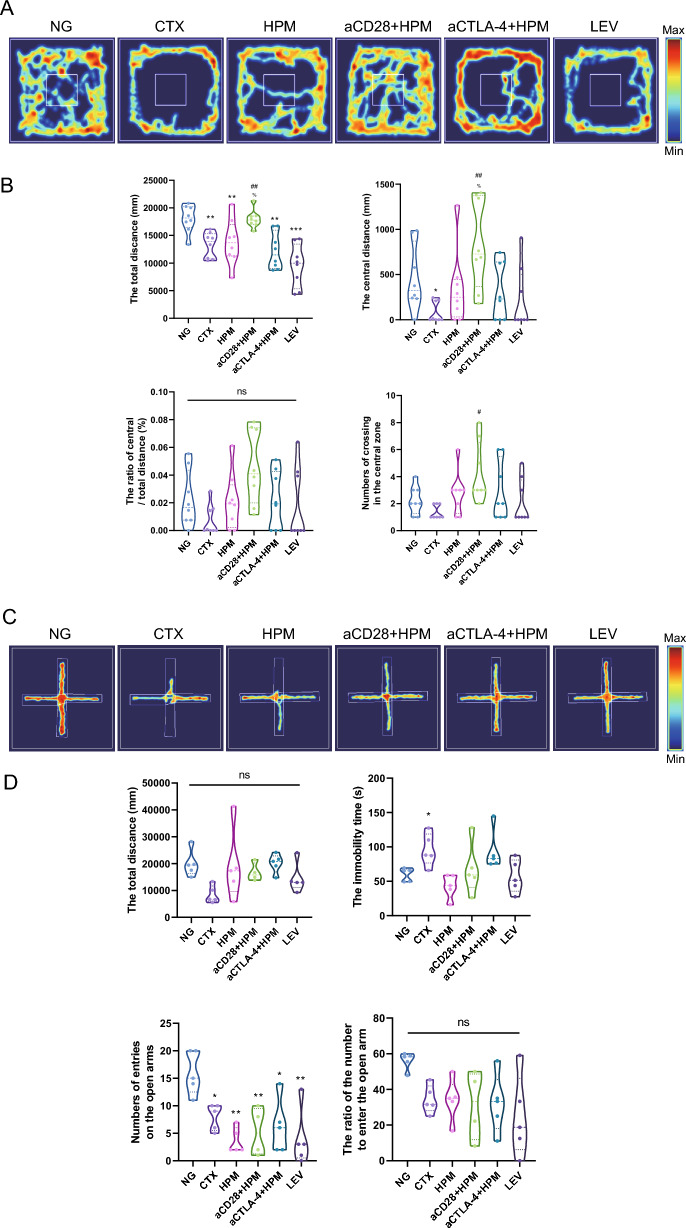
Immunosuppressed rats exhibit depressive/anxiety-like behavior, which is attenuated after HPM binding inhibitor treatment. **A**, **B** Schematic representation of OFT (n = 8 per each group). **C**, **D** Schematic representation of EPM (n = 5 per each group). Data were analyzed using *Kruskal–Wallis H-*statistic test. ^*^p < 0.05, ^**^p < 0.01vs NG. NS: no significance; OFT: open-field test; EPM: elevated plus-maze; NG: normal group; CTX: cyclophosphamide; HPM: Herb-partitioned moxibustion; aCD28: anti-CD28; aCTLA-4: anti-CTLA-4; LEV: Levamisole

At the end of all the treatments, the OFT results were observed and showed that the total distance traveled was reduced in the CTX (*p* = 0.012), HPM (*p* = 0.038), aCTLA-4 + HPM (*p* = 0.002), and LEV (*p* = 0.001) groups than in the NG group, but there was no significant difference in the aCD28 + HPM group compared to the NG group. In addition, the total distance traveled was increased for aCD28 + HPM compared to the CTX (*p* = 0.011) or HPM (*p* = 0.035) groups, which both showed a significant increase in the total distance traveled. The central distance was significantly shorter in the CTX (*p* = 0.028) group than in the NG group. In addition, compared with those in the CTX (*p* = 0.009) and HPM (*p* = 0.040) groups, the aCD28 + HPM (*p* = 0.014) group exhibited an increase in the number of crossings in the central zone compared to CTX (Fig. 2A, B).

The EPM test results revealed no difference between the groups in terms of total distance traveled or the ratio of the number of rats able to enter the open arm. Compared to those in the NG, the resting time in the CTX group (*p* = 0.037) was significantly higher. In addition, the remaining of the groups showed a trend toward a decrease in the immobility time compared to that in the CTX group, but the difference was not statistically significant. However, the immobility time of the aCTLA-4 + HPMs (*p* = 0.003) was still greater than that of the HPMs. The number of entries in the open arm was significantly lower for CTX (*p* = 0.018), HPM (*p* = 0.002), aCD28 + HPM (*p* = 0.006), aCTLA-4 + HPM (*p* = 0.019), and LEV (*p* = 0.002) than for the NG group (Fig. 2C, D).

### Effect of HPM treatment on WBC, RBC, HGB, and PLT counts in immunosuppressed rats

**Fig. 3:**
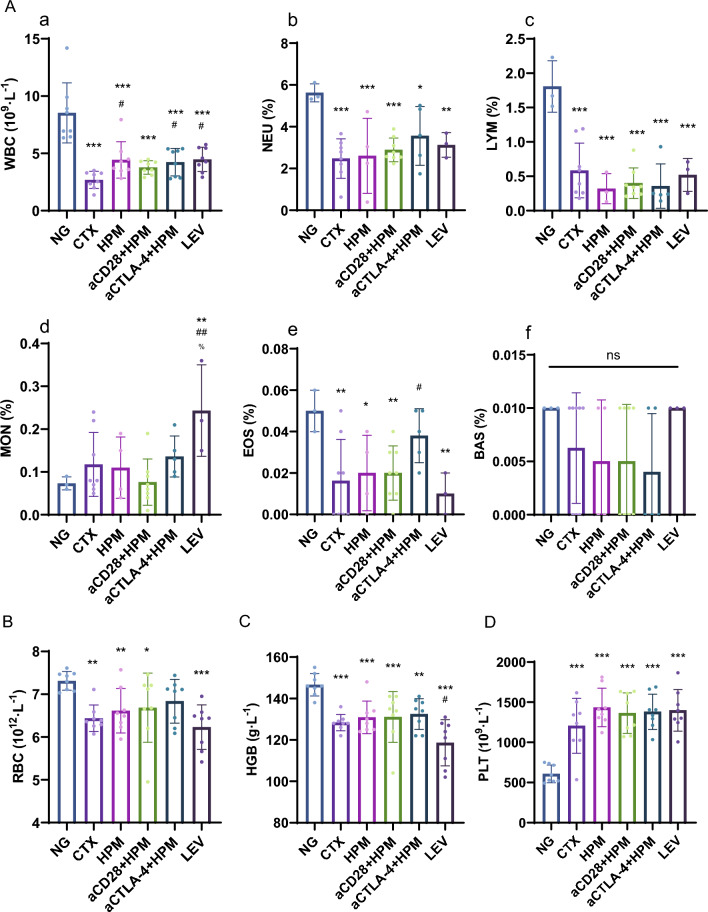
The number of WBCs was significantly reduced in rats after cyclophosphamide injection and increased after HPM treatment. **A** WBCs and their subtype percentage results. (n = 3–8 per each group). **B**–**D** RBC, hemoglobin and platelet counts (n = 8 per each group). Data were analyzed using *One-way ANOVA* tests with *LSD-t* post-hoc tests executed. ^*^p < 0.05, ^**^p < 0.01, ^***^p < 0.001vs NG; ^#^*p* < 0.05 vs CTX; ^%^*p* < 0.05 vs HPM. ANOVA: analysis of variance; LSD-t: Least Significant Difference-t, NS: no significance; NG: normal group; CTX: cyclophosphamide; HPM: Herb-partitioned moxibustion; aCD28: anti-CD28; aCTLA-4: anti-CTLA-4; LEV: Levamisole

To verify the success of the immunosuppression model, we examined blood cells, such as white blood cells (WBCs) and red blood cells (RBCs). The WBC results showed that compared to those in the NG, the CTX (*p* < 0.001), HPM (*p* < 0.001), aCD28 + HPM (*p* < 0.001), aCTLA-4 + HPM (*p* < 0.001), and LEV (*p* < 0.001) WBC counts were significantly reduced; moreover, compared to the CTX, the HPM (*p* = 0.002), aCTLA-4 + HPM (*p* = 0.041), and LEV (*p* = 0.019) were significantly increased (Fig. 3a). According to the NEU and LYM percentages, there were no significant differences between the groups after CTX injection or different treatments (Fig. 3b, c). According to the percentage of MONs, LEV was abnormally high compared to NG (*p* = 0.004), CTX (*p* = 0.009), and HPM (*p* = 0.013) (Fig. 3d).

The RBC and HGB counts results showed that CTX (*p*_*RBC*_ = 0.002, *p*_*HGB*_ < 0.001), HPM (*p*_*RBC*_ = 0.010, *p*_*HGB*_ = 0.001), aCD28 + HPM (*p*_*RBC*_ = 0.020, *p*_*HGB*_ = 0.001), and LEV (*p*_*RBC*_ < 0.001, *p*_*HGB*_ < 0.001) were reduced to varying degrees when compared with the NG (Fig. 3B, C). PLT was significantly greater in the CTX (*p* < 0,001), HPM (*p* < 0,001), aCD28 + HPM (*p* < 0,001), aCTLA-4 + HPM (*p* < 0,001), and LEV (*p* < 0,001) groups than in the NG group (Fig. 3D).

### Effect of HPM treatment on the histologic analysis of splenic injury in immunosuppressed rats

**Fig. 4:**
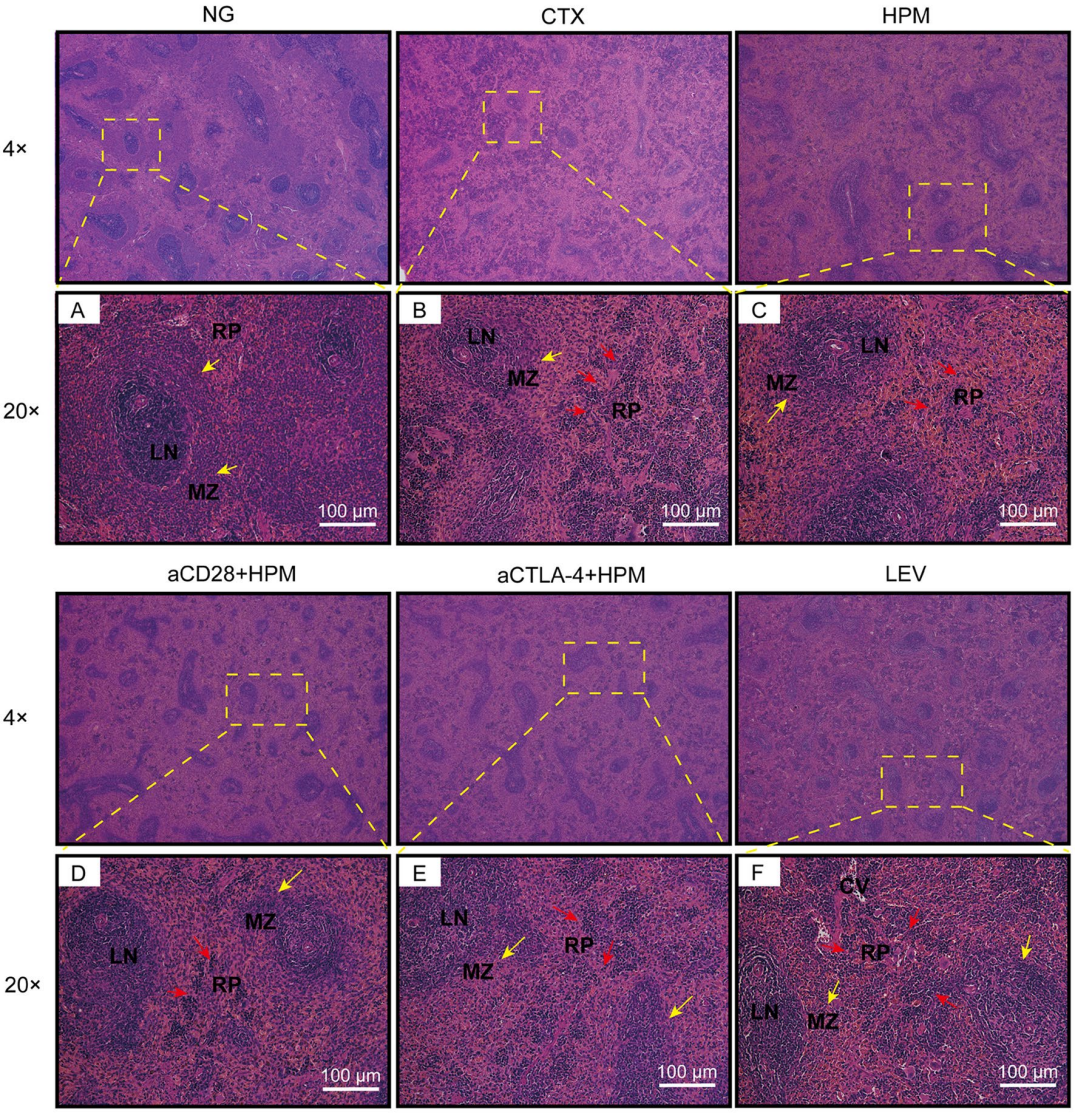
Histological analysis of spleen in immunosuppressed rats (H&E staining, bar = 100 μm). CV, central vein; LN, lymph nodule; MZ, marginal zone; RP, red pulp, NG: normal group; CTX: cyclophosphamide; HPM: Herb-partitioned moxibustion; aCD28: anti-CD28; aCTLA-4: anti-CTLA-4; LEV: Levamisole

To observe the destruction of the immune organ spleen by CTX and the effect of a treatment such as HPM, we analyzed the H&E staining of the spleen tissue. The spleen tissue in the NG (Fig. 4A) had a normal structure, no atrophy of the white marrow, an abundant marginal zone (shown by yellow arrows), and dense lymph sheath cells around the small central splenic artery. After modeling, the basic structure of the spleen tissue in the CTX was destroyed, the white marrow area was obviously reduced or even absent, the marginal zone was reduced, the number of lymph sheath cells around the small central splenic artery were reduced, additional pigmented deposits were found in the red pulp, and the number of inflammatory cells were increased (shown by red arrows) (Fig. 4B). After treatment, the marginal zone area of the spleen tissue in the HPM increased, the number of lymphatic sheath lymphocytes around the small central artery of the spleen increased, and the red pulp containing a small amount of pigmentation was partially reduced (Fig. 4C). The aCD28 + HPM and aCTLA-4 + HPM resulted in some increase in the white pulp area compared with that in the HPM group, and there was also a decrease in red pulp pigmentation compared with that in the HPM group (Fig. 4C, D), but there was still pigmentation and a large number of inflammatory cells in the red pulp of part of the splenic tissue in the LEV group (Fig. 4E).

### Effect of HPM treatment on CD28, CTLA-4, B7-1, B7-2, α-MSH, TrkB, 5-HT, and BDNF in the serum of immunosuppressed rats

To provide preliminary evidence that HPM treatment can act on immunosuppressed rats and affect both the immune system and the nervous system, we investigated relevant immune checkpoint molecules and neural-related molecules in the serum.

**Fig. 5:**
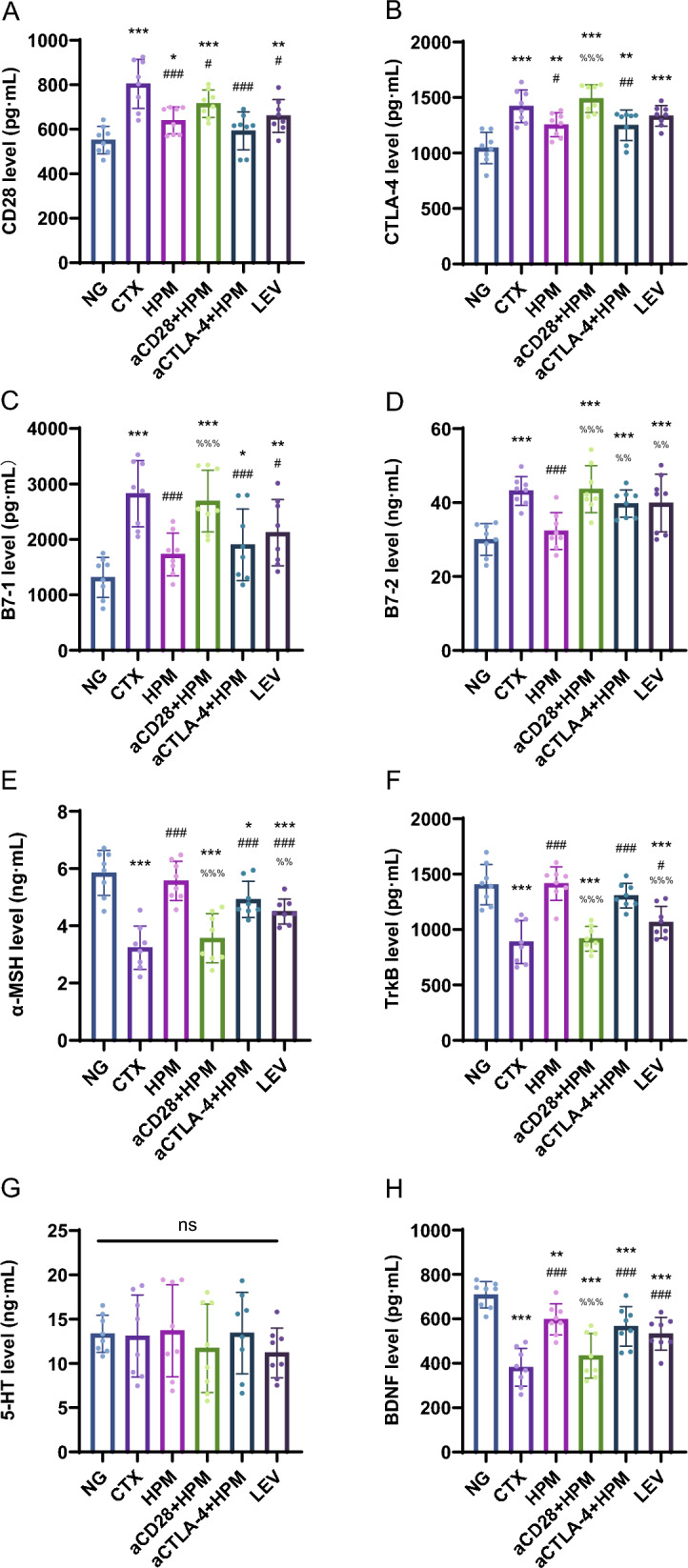
Levels of CD28 (**A**), CTLA-4 (**B**), B7-1 (**C**), B7-2 (**D**), α-MSH (**E**), TrkB (**F**), 5-HT (**G**), BDNF (**H**) in serum of immunosuppressed rats (n = 8 per each group). Data are expressed as means ± SD, *One-way ANOVA* tests with *LSD-t* post-hoc tests executed. ^*^p < 0.05, ^**^p < 0.01, ^***^p < 0.001 vs NG; ^#^*p* < 0.05, ^##^*p* < 0.01, ^###^*p* < 0.001 vs CTX; ^%%^*p* < 0.01, ^%%%^*p* < 0.001 vs HPM. α-MSH: α-Melanocyte-stimulating hormone; TrkB: Tropomyosin receptor kinase B; 5-HT: 5-hydroxytryptamine; BDNF: Brain-derived neurotrophic factor; SD: standard deviation; ANOVA: analysis of variance; LSD-t: Least Significant Difference-t, NG: normal group; CTX: cyclophosphamide; HPM: Herb-partitioned moxibustion; aCD28: anti-CD28; aCTLA-4: anti-CTLA-4; LEV: Levamisole

In the serum results of immune checkpoint molecules, compared to the NG group, the CTX(all *p* < 0.001), aCD28 + HPM (all *p* < 0.001), and LEV (*p*_CD28_ = 0.08, *p*_CTLA-4_ < 0.001, *p*_B7-1_ = 0.004, *p*_B7-2_ = 0.001) groups significantly increased in CD28, CTLA-4, B7-1, and B7-2; in the aCTLA-4 + HPM group, there was an increase in CTLA-4, B7-1, and B7-2 (*p*_CTLA-4_ = 0.02, *p*_B7-1_ = 0.033, *p*_B7-2_ = 0.001); the HPM group increased only in CD28 and CTLA-4 (*p*_CD28_ = 0.028, *p*_CTLA-4_ = 0.002). Compared to the CTX group, the HPM group (all *p* < 0.01) significantly decreased in CD28, CTLA-4, B7-1, and B7-2; the aCTLA-4 + HPM gourp (all *p* < 0.01) significantly decreased in CD28, CTLA-4, and B7-1; the LEV group (*p*_CD28_ = 0.001, *p*_B7-1_ = 0.012) decreased in CD28 and B7-1; the aCD28 + HPM group (*p* = 0.026) decreased only in CD28. Compared to the HPM group, the aCD28 + HPM group (all *p* ≤ 0.001) significantly increased in CTLA-4, B7-1, and B7-2; the aCTLA-4 + HPM (*p* = 0.009) and LEV (*p* = 0.008) groups increased only in B7-2 (Fig. 5A–D).

In the serum results of neuro-related molecules, serotonin showed no statistically significant differences between groups. Compared to the NG group, the CTX (all *p* < 0.001), aCD28 + HPM (all *p* < 0.001), and LEV (all *p* < 0.001) groups significantly decreased in α-MSH, TrkB, and BDNF; the aCTLA-4 + HPM group (p_α-MSH_ = 0.012, p_BDNF_ = 0.001) decreased in α-MSH and BDNF; the HPM group (*p* = 0.009) decreased only in BDNF. Compared to the CTX group, the HPM (all *p* < 0.001), aCTLA-4 + HPM (all *p* < 0.001), and LEV (*p*_α-MSH_ = 0.001, *p*_TrkB_ = 0.024, *p*_BDNF_ = 0.001) groups decreased in α-MSH, TrkB, and BDNF levels. Compared to the HPM group, the aCD28 + HPM group (all *p* < 0.001) significantly increased in α-MSH, TrkB, and BDNF; LEV (*p*_α-MSH_ = 0.004, *p*_TrkB_ < 0.001) increased only in α-MSH and TrkB (Fig. 5E–H).

### *Effect of HPM treatment on CD*_*4*_^+^*and CD*_*8*_^+^*T cells in the spleens of immunosuppressed rats*

**Fig. 6:**
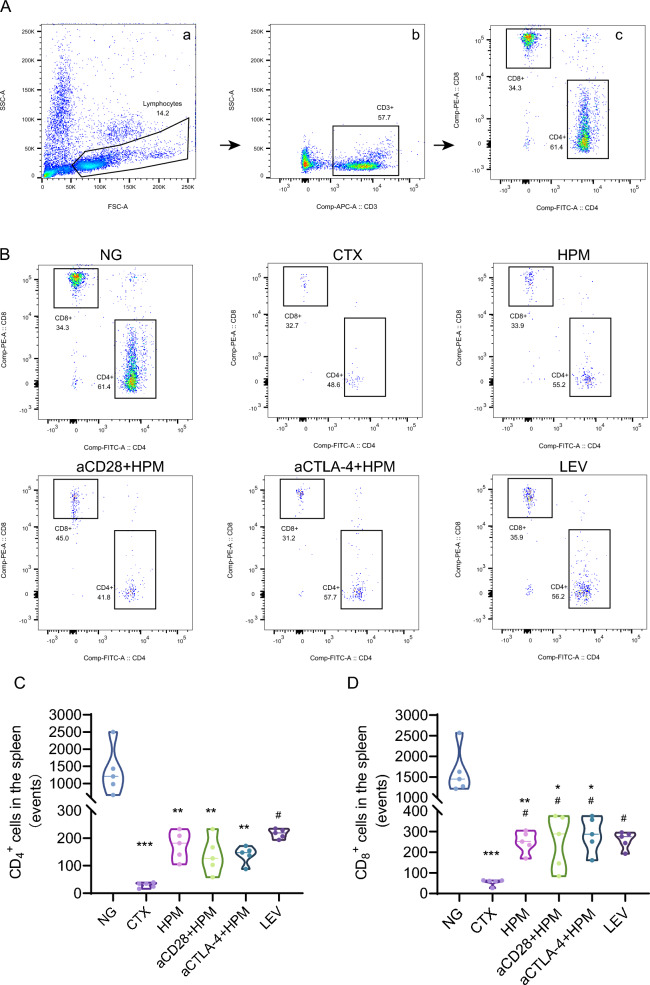
Effect of HPM treatment on T lymphocytes in the spleens of immunosuppressed rats. **A** CD4+, CD8+ cells were gated by the protocol. **B** Flow cytometric identification of CD4 + , CD8 + cells in the spleen. **C**, **D** Absolute count statistics of CD4+, CD8+ cells between different groups in spleen tissue (n = 5 per each group). Data were analyzed using *Kruskal–Wallis H-statistic* test. **p < 0.01, ***p < 0.001vs NG; ^#^*p* < 0.05 vs CTX. NG: normal group; CTX: cyclophosphamide; HPM: Herb-partitioned moxibustion; aCD28: anti-CD28; aCTLA-4: anti-CTLA-4; LEV: Levamisole

To determine the putative effect of HPM treatment on T cells, we examined lymphocyte expression in spleen tissue. Flow cytometry was used to determine the absolute numbers of CD_3_^+^, CD_4_^+^, and CD_8_^+^ lymphocytes in the treated rats (the gating strategy is shown in Fig. 6A). The results for the CD_4_^+^ cell counts showed that, compared to the NG, the CTX (*p* < 0.001), HPM (*p* = 0.036), aCD28 + HPM (*p* = 0.005), and aCTLA-4 + HPM (*p* = 0.005) were significantly reduced. Only LEV (*p* = 0.018) had a significant increase compared to CTX, and HPM and aCTLA-4 + HPM tended to increase compared to CTX, but not significantly (Fig. 6B, C). The results for the CD_8_^+^ cell counts were significantly lower in the CTX (*p* < 0.001), HPM (*p* = 0.013), aCD28 + HPM (*p* = 0.030), and aCTLA-4 + HPM (*p* = 0.046) groups than in the NG group. Compared to those of CTX, the HPM (*p* = 0.044), aCD28 + HPM (*p* = 0.020), aCTLA-4 + HPM (*p* = 0.013), and LEV (*p* = 0.031) groups were significantly increased (Fig. 6B, D).

### Effect of HPM treatment on CD28, CTLA-4, B7-1, and B7-2 in the spleens of immunosuppressed rats

**Fig. 7:**
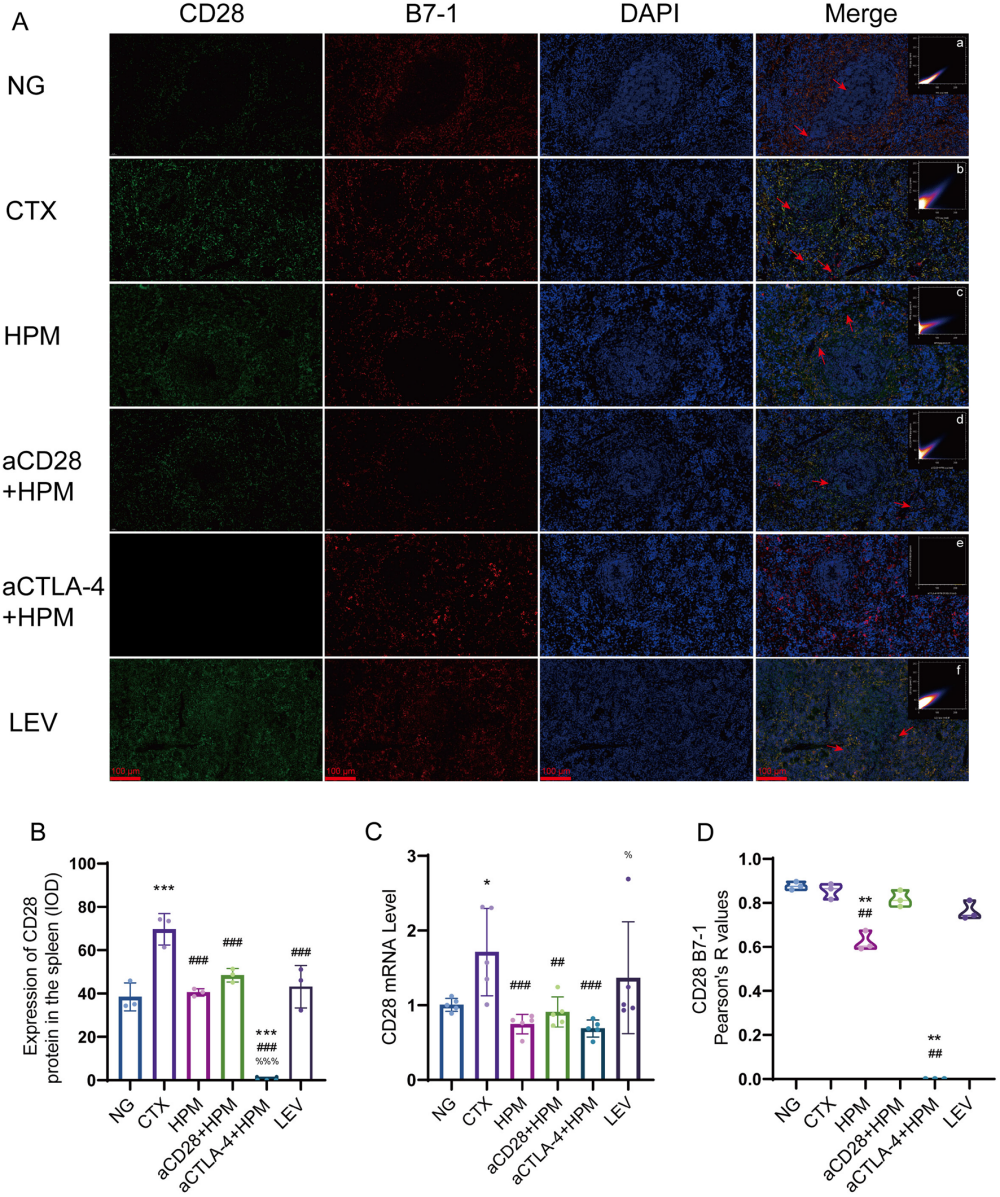
Results of the CD28 mRNA level and correlation between CD28 and B7-1 in spleen of immunosuppressed rats (n = 3–5 per each group). **A** immunofluorescence results, **a**–**f** *Pearson* correlation scatterplot; **B** CD28 IOD results; **C** CD28 mRNA level; **D** CD28 and B7-1 colocalization results. Data are expressed as means ± SD, *One-way ANOVA* tests with *LSD-t* post-hoc tests executed and *Pearson* correlation coefficient, *Kruskal–Wallis one-way ANOVA* test. ^*^p < 0.05, ^**^p < 0.01, ^***^p < 0.001 vs NG; ^##^*p* < 0.01, ^###^*p* < 0.001 vs CTX; ^%^*p* < 0.05, ^%%%^*p* < 0.001 vs HPM. IOD: integrated optical density; SD: standard deviation; ANOVA: analysis of variance; LSD-t: Least Significant Difference-t, NG: normal group; CTX: cyclophosphamide; HPM: Herb-partitioned moxibustion; aCD28: anti-CD28; aCTLA-4: anti-CTLA-4; LEV: Levamisole

HPM can effectively inhibit the expression of CD28 and CTLA-4. Immunofluorescence analysis revealed that, compared with those in the NG group, the CD28 levels in the CTX group were significantly higher (*p* < 0.001), while the HPM, aCD28 + HPM, and LEV groups were not significantly different (all *p* > 0.05). Furthermore, compared with those in the CTX group, the CD28 content in the treatment groups was significantly lower (all *p* < 0.001). No significant changes were observed in the other treatment groups compared to the HPM group (all *p* > 0.05), and no data was available for the aCTLA-4 + HPM group (Fig. 7B). Further RT-qPCR analysis (Fig. 7C) revealed that the CD28 mRNA level in spleen tissues was significantly higher in the CTX (*p* = 0.011) than in the NG group. Compared with that in the CTX, the CD28 mRNA level was significantly lower in the HPM (*p* = 0.001), aCD28 + HPM (*p* = 0.004), and aCTLA-4 + HPM (*p* = 0.001) groups. Compared to the HPM, CD28 mRNA levels were significantly higher in the LEV (*p* = 0.023).

In terms of CTLA-4 analysis, compared to the NG group, there was a significant increase in CTLA-4 content in the CTX group (*p* = 0.002), while no significant changes were observed in the HPM, aCTLA-4 + HPM or LEV groups (all *p* > 0.05). Additionally, the treatment groups demonstrated a significant decrease in CTLA-4 content compared to the CTX group (all *p* < 0.01). Compared to the HPM group,the aCTLA-4 + HPM group had a decrease in CTLA-4 (*p* = 0.045),and no data were available for the aCD28 + HPM group (Fig. 9B). Further RT-qPCR analysis (Fig. 9C) revealed that CTLA-4 mRNA levels in spleen tissues was significantly higher in the CTX (*p* < 0.001), but significantly lower in the HPM (*p* = 0.021), aCD28 + HPM (*p* = 0.009), and aCTLA-4 + HPM (*p* < 0.001) compared with that in the NG group. CTLA-4 mRNA levels were significantly lower in the HPM (*p* = 0.001), aCD28 + HPM (*p* = 0.004) and aCTLA-4 + HPM (*p* = 0.001) compared to that in the CTX. Compared to the HPM, the CTLA-4 mRNA level was significantly lower in the aCTLA-4 + HPM (*p* = 0.006). The PCR results for B7-1 (Fig. 9D) showed that the B7-1 mRNA levels in spleen tissues were significantly higher in the CTX (*p* < 0.001), aCD28 + HPM (*p* < 0.001) and LEV (*p* = 0.003) groups compared with the NG group. Compared with that in the CTX, the B7-1 mRNA levels were significantly lower in the HPM (*p* = 0.002) and aCTLA-4 + HPM (*p* = 0.005). Compared to the HPM, the B7-1 mRNA levels were significantly higher in the aCD28 + HPM (*p* = 0.007).

**Fig. 8:**
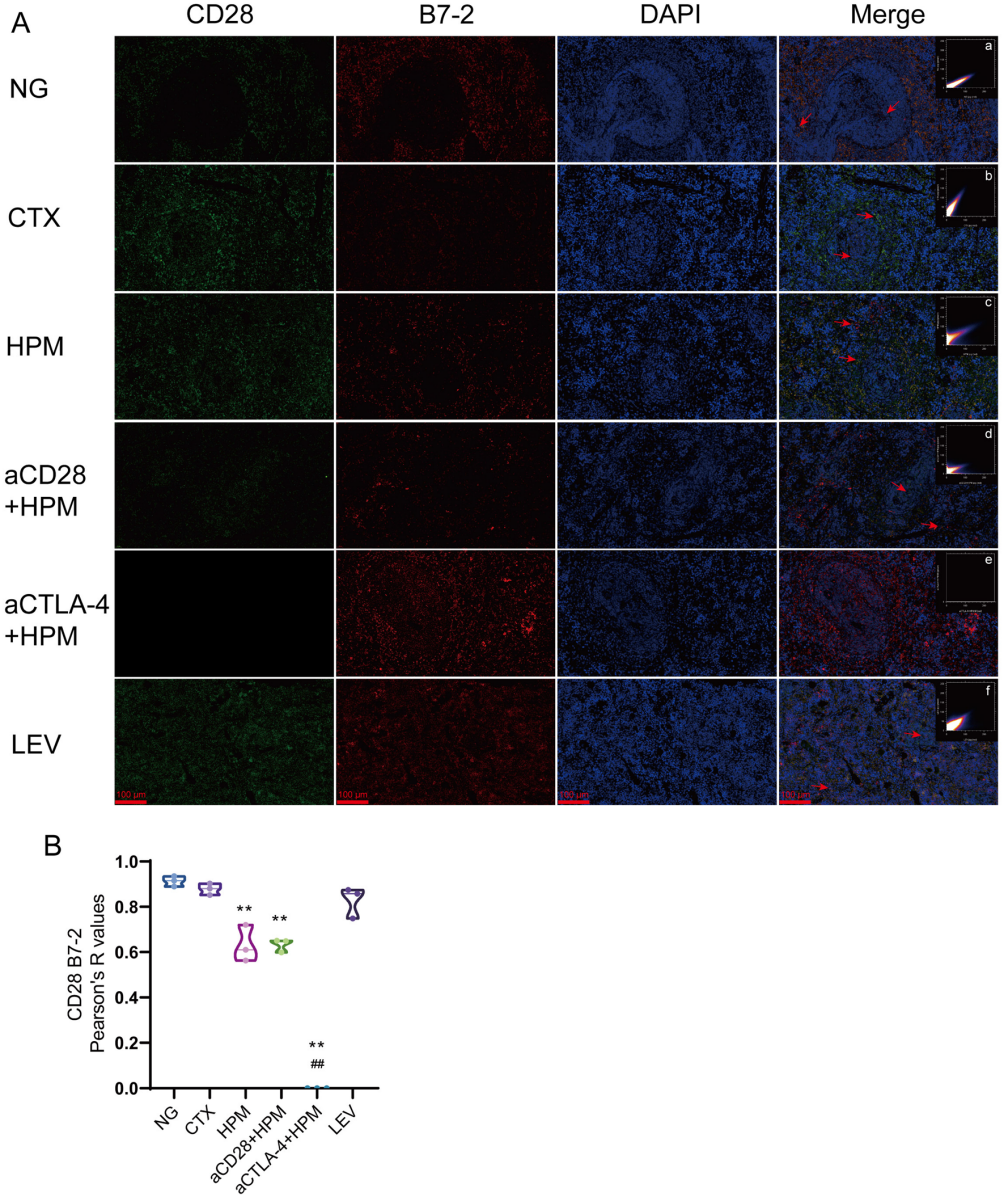
Results of the correlation between CD28 and B7-2 in spleen of immunosuppressed rats (n = 3 per each group). **A** immunofluorescence results, **a**–**f** *Pearson* correlation scatterplot; **B** CD28 and B7-2 colocalization results. Data are expressed as *Pearson* correlation coefficient, *Kruskal–Wallis one-way ANOVA* test. ^**^p < 0.01 vs NG; ^##^*p* < 0.01 vs CTX. NG: normal group; CTX: cyclophosphamide; HPM: Herb-partitioned moxibustion; aCD28: anti-CD28; aCTLA-4: anti-CTLA-4; LEV: Levamisole

CTLA-4 combined with B7-1 and B7-2, CD28 combined with B7-1 and B7-2 had different degrees of colocalization in the spleen. The colocalization was analyzed and plotted by ImageJ. The results of CD28 and B7-1 colocalization (Fig. 7A, a–f,  7D) showed that the Pearson coefficients of the NG group and CTX group were (*r* = 0.87, *r* = 0.84), the Pearson coefficients of the HPM and NG groups were decreased (*r* = 0.62, *p* = 0.011), and the Pearson coefficients of the HPM and CTX groups were decreased (*r* = 0.62, *p* = 0.026). The results of CD28 and B7-2 colocalization (Fig. 8A, a–f,  8B) showed that the Pearson coefficients of the NG and CTX groups were (*r* = 0.91, *r* = 0.88), the Pearson coefficients of the HPM and NG groups were decreased (*r* = 0.64, *p* = 0.018), and the Pearson coefficients of the aCD28 + HPM and NG groups were decreased (*r* = 0.57, *p* = 0.022).

**Fig. 9:**
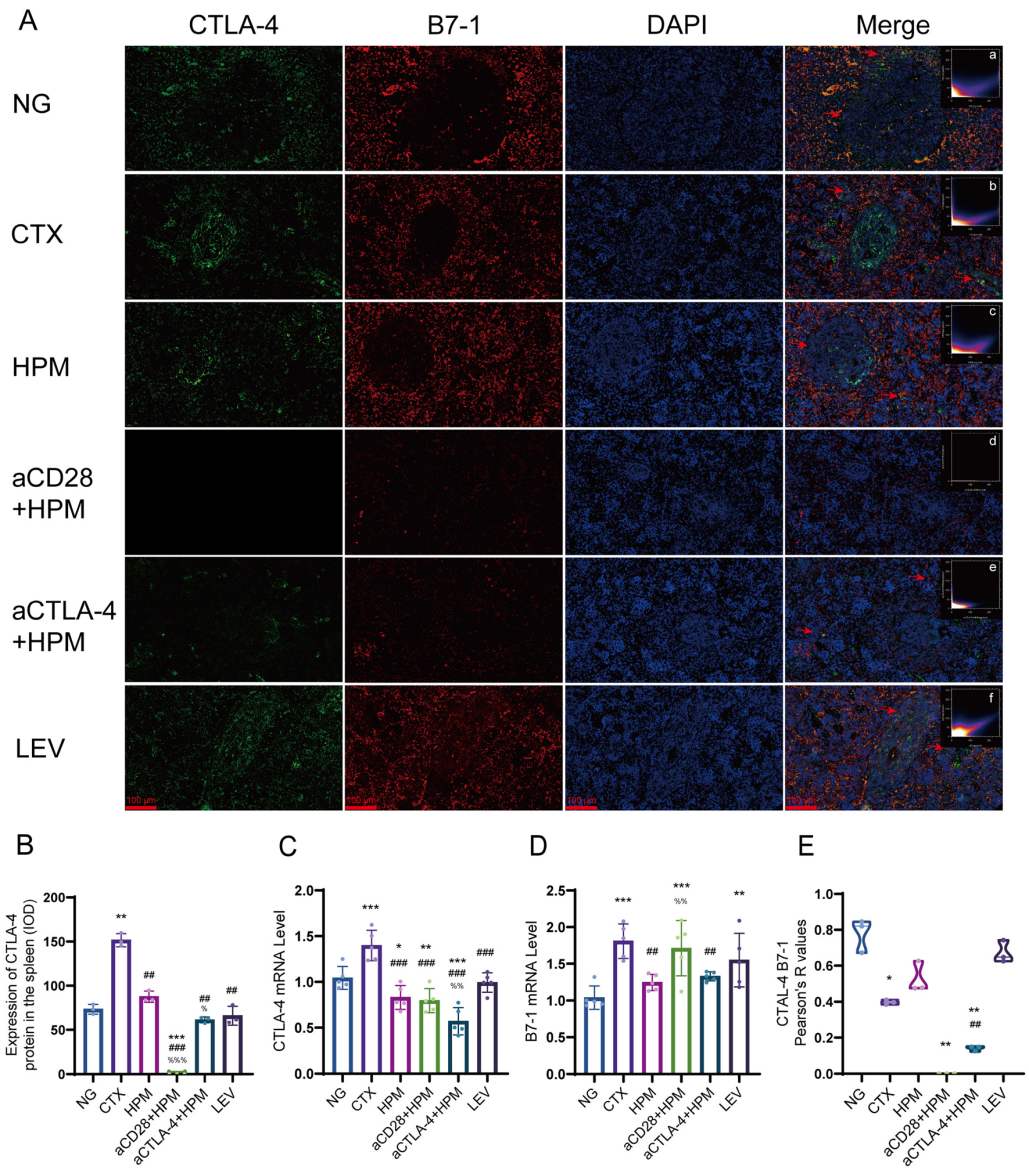
Results of CTLA-4 mRNA level and the correlation between CTLA-4 and B7-1 in spleen of immunosuppressed rats (n = 3–5 per each group). **A** immunofluorescence results, **a**–**f** *Pearson* correlation scatterplot; **B** CTLA-4 IOD results; **C** CTLA-4 mRNA level; **D** B7-1 mRNA level; **E** CTLA- and B7-1 colocalization results. Data are expressed as means ± SD, *One-way ANOVA* tests with *LSD-t* post-hoc tests executed and *Pearson* correlation coefficient, *Kruskal–Wallis one-way ANOVA* test. ^*^p < 0.05, ^**^p < 0.01, ^***^p < 0.001 vs NG; ^##^*p* < 0.01, ^###^*p* < 0.001 vs CTX; ^%%%^*p* < 0.001 vs HPM. IOD: integrated optical density; SD: standard deviation; ANOVA: analysis of variance; LSD-t: Least Significant Difference-t, NG: normal group; CTX: cyclophosphamide; HPM: Herbpartitioned moxibustion; aCD28: anti-CD28; aCTLA-4: anti-CTLA-4; LEV: Levamisole

**Fig. 10:**
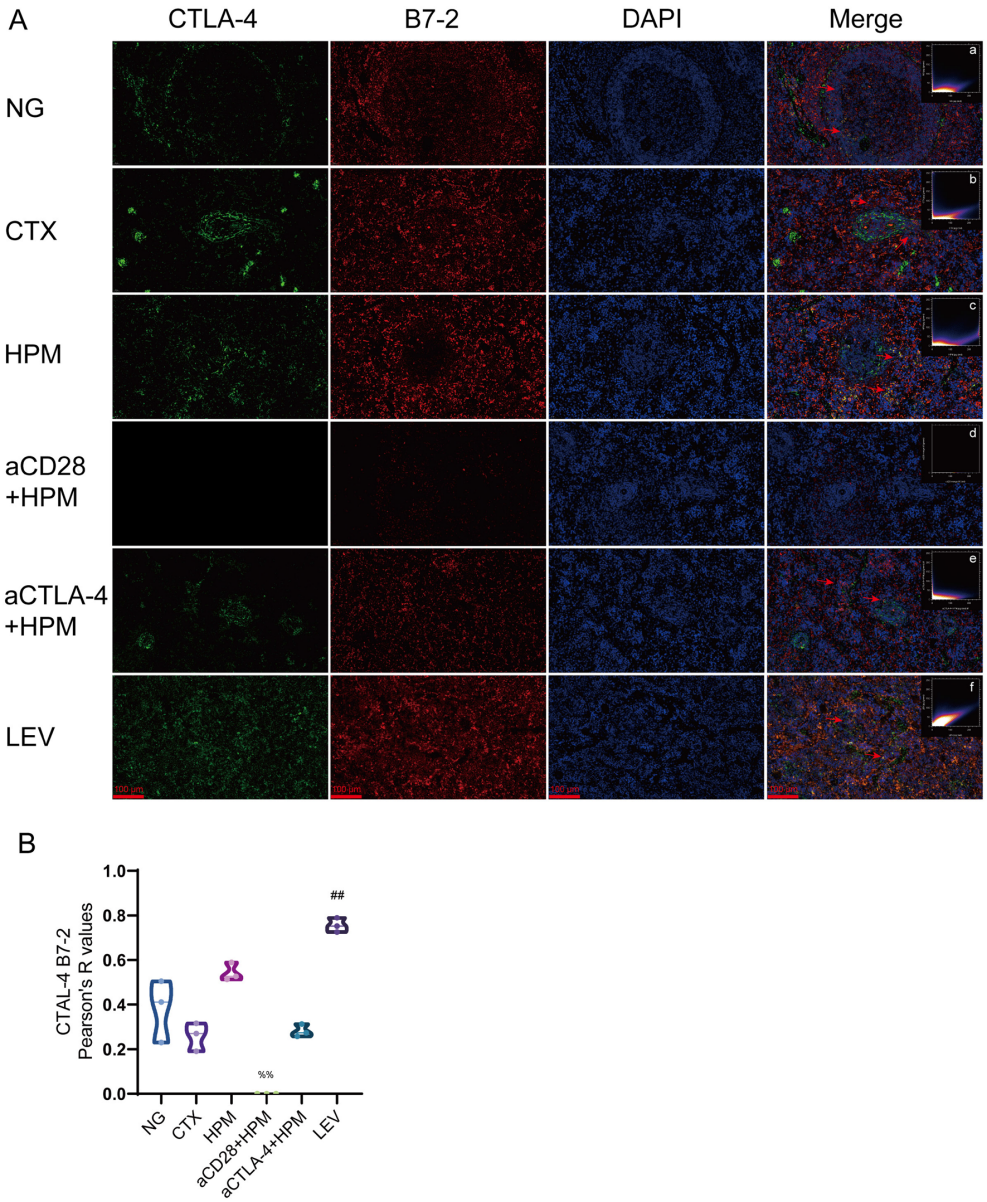
Results of the correlation between CTLA-4 and B7-2 in spleen of immunosuppressed rats (n = 3 per each group). **A** immunofluorescence results, **a**–**f** *Pearson* correlation scatterplot; **B** CTLA-4 and B7-2 colocalization results. Data are expressed as *Pearson* correlation coefficient, *Kruskal–Wallis one-way ANOVA* test. ^##^*p* < 0.01 vs CTX; ^%%^*p* < 0.01 vs HPM. NG: normal group; CTX: cyclophosphamide; HPM: Herb-partitioned moxibustion; aCD28: anti-CD28; aCTLA-4: anti-CTLA-4; LEV: Levamisole

The results of CTLA-4 and B7-1 colocalization (Fig. 9A, a–f, 9E) showed that the Pearson coefficient of the NG group was (*r* = 0.78), the Pearson coefficients of the CTX and NG groups were decreased (*r* = 0.40, *p* = 0.046), and the Pearson coefficients of the aCTLA-4 + HPMand NG groups were decreased (*r* = 0.014, *p* = 0.007). The results of CTLA-4 and B7-2 colocalization (Fig.10A, a–f, 10B) showed that the Pearson coefficients of the NG and CTX groups were (*r* = 0.38, *r* = 0.26), and only the LEV and CTX groups had increased Pearson coefficients (*r* = 0.75, *p* = 0.022).

## Discussion

The immunosuppressive state caused by the failure of T cells plays an improtant role in immune deficiency and the development of suboptimal health [1, 2], as it not only strongly impairs the quantity and quality of lymphocytes but also has a pronounced impact on the generation of inflammatory cells and emotional well-being [32, 33].

Previous studies on immune suppression have indicated that acupuncture can effectively ameliorate the inflammatory environment and exert a regulatory effect on emotions [34, 35]. However, there is still a lack of clarity understanding regarding the impact of acupuncture, particularly moxibustion, on the failure of T-cell activity under immune-deficient conditions. In this study, we found that moxibustion, especially HPM, can promote an increase in the number of T cells and alleviate inflammation through the combined effects of moxibustion, acupoints, and Chinese medicinal herbs. Increasing evidence suggests that acupuncture regulates the body’s immune system through multiple targets and pathways [36, 37]; however, previous studies have focused mostly on specific pathways or singular organ effects. Building upon prior research, this study revealed that, from a neuroimmune perspective, moxibustion can restore immune function in immunosuppressed rats by promoting T-cell activation pathways and inhibiting T-cell suppressive pathways. The acupuncture points utilized in this study, namely, ST36 [24], CV4 [18], CV8 [23], and CV12 [27], have demonstrated the ability to enhance the immune response. The selection of levamisole as a control group was based on previous research conducted by Sajad [38] and Lekhooa [31].

Recent reports on immune suppression have revealed that there is a notable decrease in body weight following CTX injection, and this decrease persists for a prolonged period, which is corroborated by our data [39, 40]. Moreover, we observed that rapid intervention after model establishment effectively mitigated the occurrence of weight loss, indicating that the thermal stimulation via the HPM may protect the body from the adverse effects of CTX. However, interestingly, the aCTLA-4 + HPM group did not exhibit a noticeable increase in body weight, which might be associated with the modulation of these proteins by the inhibitors. The exact underlying mechanisms are currently unclear and warrant further research.

Notably, in this study, compared to those in the NG group, the spleen indices in the CTX group were significantly lower (Fig. 1E), which contradicts the findings of Lv [40], but aligns with the findings of Zhang, Nam, et al. [41, 42]. These starkly contrasting findings suggest the occurrence of differential immune disruption caused by CTX in different animal species, while the timing of the establishment of the immunosuppression model using cyclophosphamide is one of the key factors in the variability. However, there is currently a lack of comprehensive reports on how CTX precisely affects different experimental animals, and we have decided to further investigate this topic in the next phase of our research.

Behavioral observations are typically used to assess the depressive state of animals. However, we were inspired by Dougherty’s research [43] to evaluate the activity level of immunosuppressed rats through the OFT and EPM test to observe whether HPM can ameliorate the impaired activity performance of immunosuppressed rats. In this study, it was observed that in the OFT, there was a significant reduction in the total distance traveled by rats following CTX intervention compared to that traveled by normal rats. In the EPM test, the CTX group exhibited significantly longer immobility times than did the other groups, indicating a pronounced decrease in the physical activity of immunosuppressed rats. Indeed, prior research [44] on the connection between immune function and physical activity has indicated that CTX can induce muscle damage while compromising the immune system, consequently impacting the body’s motor function. This study’s behavioral assessments produced analogous findings (Fig. 2). A more comprehensive validation of the CTX model demonstrated that WBC counts in immunosuppressed rats were notably lower than those in the NG group, remaining at 2.68 × 10^9^/L, with NEU% also being significantly lower than that in the NG group. Following HPM treatment, there was an overall increase in WBC count, indicating that HPM has the capacity to stimulate an increase in white blood cells (Fig. 3Aa). As described above, HPM treatment can improved the quantity of WBCs and reduced the immobility time of immunosuppressed rats, enhancing their physical activity.

The spleen, recognized as a vital immune organ, harbors diverse lymphatic cells. Its principal structural elements include the white pulp (WP), which is responsible for blood filtration, antigen clearance, and the removal of senescent red blood cells; the red pulp (RP), which is associated with lymphatic cell generation and involvement in immune responses; and the marginal zone (MZ), which is capable of producing cytokines and chemokines following immune cell activation. Further observation of the histological structure of the spleen in immunosuppressed rats revealed that the boundaries of the WP and RP in the NG group were clear, with a visible intact MZ. In contrast, in the CTX group, the white pulp area was reduced, and the MZ region was significantly damaged, with a noticeable presence of inflammatory cells. After intervention with HPM and various inhibitors, the WP area was restored, and an increase in WP indicated an improvement in immune function [45]. Ouyang’s study has likewise affirmed that TCM therapies such as moxibustion can act against aging through immune system regulation [18]. This finding is consistent with the outcomes of our research group’s previous findings. Therefore, based on the H&E staining results, it can be concluded that HPM, by restoring the WP region, restores the body’s immune system (Fig. 4C).

By combining routine blood and H&E staining results, it is evident that the HPM effectively restores white blood cell counts, rehabilitates the spleen’s WP structure, and ameliorates the functional decline resulting from immunosuppression. Previous research by our project team has shown that HPM can enhance immune function by modulating the levels of the immune checkpoint PD-1. Therefore, in this study, we aimed to explore whether the regulatory effects of HPM on immune checkpoints involve multiple pathways. Among the immune regulatory agents, CD28 and CTLA-4 positively and negatively regulate T-cell proliferation, respectively [46]. In this study, we inhibited CD28 and CTLA-4 separately and assessed the binding ability of another molecule, the B7-1 and B7-2 receptors they share, to investigate the regulatory effects of HPM on CD28 and CTLA-4. The results showed that the trends in the levels of CD28, CTLA-4, B7-1, and B7-2 were similar across all groups, with levels in the CTX group being greater in the blood than those in the NG group (Fig. 5A–D). In the HPM group, these levels exhibited varying degrees of decrease, indicating that HPM has a certain inhibitory effect on the markedly elevated CD28 and CTLA-4 levels. A particularly intriguing observation is that, following the inhibition of CD28 expression, elevated CD28 levels remained detectable in the serum. This phenomenon could be attributed to the role of CTX in bone marrow suppression, wherein, despite the administration of CD28 inhibitors, bone marrow proliferation occurs, giving rise to CD28-positive T cells. It is also possible that unsuppressed CD28-positive cell populations, such as NK cells, exist, thus suggesting that source of CD28 may need to be verified through cellular differentiation and related methods. Nonetheless, it is essential to consider the possibility that the impact of moxibustion on the costimulatory molecule CD28 may be mediated by alternative regulatory mechanisms.

After conducting behavioral observations of immunosuppressed rats, we analyzed proteins such as α-MSH, TrkB, 5-HT, and BDNF, which play crucial roles in the nervous and endocrine systems, in order to investigate whether there is a connection between depressive emotions and immunosuppression. α-MSH is a neuropeptide, and past studies have indicated its association with depression [47] and its ability to induce immunosuppressive Tregs [48]. TrkB is a receptor for neurotrophic growth factors and has also been found to be related to depression [49]. Furthermore, another study has indicated that long-term exposure to cyclophosphamide results in persistent inhibition of BDNF expression and the display of pronounced anxiety-like behaviors [50], findings that align with what we have previously observed. In conjunction with research on 5-HT, it has been found that alterations in 5-HT levels are not solely linked to emotional states but also strongly correlated with fatigue levels [51]. Our results indicate that the reduction in total distance covered by rats and the increase in immobility time caused by CTX may be related to depressive emotions rather than fatigue. Further analysis led to the speculation that the regulatory effects of HPM on immunosuppression and depression may be connected to central nervous system regulation and sympathetic nervous system stimulation (Fig. 5E–H).

Returning to the study on immune checkpoints, the flow cytometry results of CD_4_^+^ and CD_8_^+^ T lymphocytes in spleen tissue (Fig. 6), combined with the results of WBC (Fig. 3a), affirmed that the absolute values of lymphocytes have decreased, indicating the successful establishment of an immunosuppressed rat model. In contrast, the results of absolute counts of CD_4_^+^ and CD_8_^+^ cells significantly increased in the different treatment groups. Hence, we conclude that HPM can restore immune function by replenishing CD_4_^+^ and CD_8_^+^ lymphocytes, with CD_8_^+^ lymphocytes demonstrating a superior effect compared to tha of CD_4_^+^ T lymphocytes.

CD28 and CTLA-4 are both crucial transmembrane molecules on T lymphocytes. To better understand the regulatory effect of HPM on immune function, we individually inhibited CD28 and CTLA-4 and observed the differences in spleen tissue among the different treatment groups. The results revealed that CD28 is expressed at a very low level in the spleen tissues of normal rats. After CTX stimulation, there is high CD28 expression in the WP and RP regions [52]. Interestingly, in the HPM and aCD28 + HPM groups, after 10 days of treatment, the structure of the MZ area at the periphery of the WP had partially recovered [53], and the CD28 levels decreased (Fig. 7B). PCR for CD28 mRNA (Fig. 7C) showed the same results. However, in conjunction with the ELISA results, CD28 was expressed at lower levels in the spleen tissues of the CTX group but a higher levels in the serum. The possible reason for this difference is that cyclophosphamide inhibits the internalization process of immune cells toward CD28, causing a large quantity of membrane-bound CD28 molecules to enter the bloodstream. This requires further in-depth research for verification. Furthermore, CTLA-4 is predominantly expressed in the RP and MZ regions in the spleen tissues of normal rats. After CTX injection, CTLA-4 expression in the spleen shifted from the RP to the WP. Following treatment with HPM or other agents, the reduction in CTLA-4 in the WP was significant, and its expression level in the spleen also decreased. The results of the PCR assay for CTLA-4 mRNA (Fig. 9C) support this conclusion. Interestingly, CD28 engagement with B7-1 on antigen-presenting cells promotes T cell activation, proliferation, and differentiation. CD28 inhibitors in the aCD28 + HPM group abrogated this costimulatory signal by blocking CD28 binding to B7-1 and B7-2, leading to T cell hypoactivity. To compensate for reduced T cell stimulation, APCs may upregulate B7-1 expression, potentially contributing to the significantly higher B7-1 mRNA levels observed in the aCD28 + HPM group compared to other treatment groups (Fig. 9D). Upon further observation of the colocalization of CD28 with B7-1 and B7-2 in each group, it was evident that the colocalization between CD28 and B7-1 weakened only in the HPM group, whereas the colocalization of CD28 with B7-2 diminished in both the HPM group and the aCD28 + HPM group (Figs. 7D, 8B). After 10 days of treatment, T lymphocyte proliferation likely entered a stable phase.

A limitation of this study is that we exclusively examined the differential effects of HPM treatment on CD28 + and CTLA-4 + cells in the spleen without conducting an in-depth exploration of the entire regulatory pathway. Currently, the available literature on the positive impact of moxibustion on the human immune system remains limited. To deepen our understanding and substantiate the connection between moxibustion and immunity, further exploration is warranted. This entails not only investigating the regulatory mechanisms of the brain-spleen axis in immune and emotional regulation but also formulating more methodologically sound clinical trials to observe moxibustion’s regulatory effects on the human body. These aspects constitute the focus of our research group’s forthcoming investigations.

## Conclusion

In conclusion, our study suggested that HPM can regulate the activity of CD_4_^+^ and CD_8_^+^ T lymphocytes through the different pathways involving of CD28 and CTLA-4, thereby restoring the immune function of immunosuppressed rats and, to some extent, improving depressive emotions under immunosuppressive conditions.

## Data Availability

Not applicable.

## Abbreviations

CTX: Cyclophosphamide
HPM: Herb-partitioned moxibustion
NG: Normal group
α-MSH: α-Melanocyte-stimulating hormone
TrkB: Tropomyosin receptor kinase B
BDNF: Brain-derived neurotrophic factor
5-HT: 5-Hydroxytryptamine
CTLA-4: Cytotoxic T-lymphocyte associated protein 4
MyD88: Myeloid differentiation primary response protein 88
WBC: White blood cells
NEU: Neutrophil
LYM: Lymphocyte
MONO: Monocyte
EOS: Eosinophils
BASO: Basophil
RBC: Red blood cells
HGB: Hemoglobin
HCT: Hematocrit
PBS: Phosphate buffered saline
BSA: Bovine serum albumin
OFT: Open field test
EPM: Elevated plus maze
H&E: Hematoxylin and eosin
WP: White pulp
RP: Red pule
MZ: Marginal zone
APC: Antigen-presenting cell
TCM: Traditional Chinese medicine
DAPI: 6-Diamidino-2-phenylindole
